# Physiology: A perspective from the Australian experience

**DOI:** 10.14814/phy2.70045

**Published:** 2024-09-09

**Authors:** Robyn Murphy, Séverine Lamon, Livia Hool

**Affiliations:** ^1^ Department of Biochemistry and Chemistry, School of Agriculture, Biomedicine and Environment La Trobe University Melbourne Victoria Australia; ^2^ Centre for Sex and Gender Equity in Health and Medical Research Deakin University Burwood Victoria Australia; ^3^ School of Human Sciences The University of Western Australia Crawley Western Australia Australia; ^4^ Victor Chang Cardiac Research Institute Darlinghurst New South Wales Australia

Australian medical researchers are internationally recognized for their significant contributions to the medical sector, spanning from preclinical fundamental discoveries to large multicenter clinical trials and public health policies. Examples of Australian ingenuity include the design of the cardiac pacemaker, the cochlear implant, a cervical cancer vaccine, and the identification of Helicobacter as a cause of gastric ulcers. Howard Florey's discovery of penicillin is estimated to have saved over 500 million lives worldwide (https://www.path.ox.ac.uk/centenary/our‐history/). None of these discoveries would have been possible without a fundamental understanding of human physiology. The discipline of physiology provides the building blocks to understand health and disease, integrating from the molecular and cellular to the tissue and whole organ. It underpins the foundations of pharmacology, biochemistry, cell biology, and medicine.

The Australian Physiological Society (AuPS) was founded in 1960 after discussions in 1957 between some of our renowned physiologists from across Australia. Notably, the records indicate these were all men and it is unclear whether any female physiologists were involved in the original discussions. The first scientific meeting of AuPS was held in 1960 and meetings were held twice per year until the early 2000s, when they became annual (http://aups.org.au/About/#history). Several years after the first meeting, in 1967, the society was named the Australian Physiological and Pharmacological Society (APPS) in recognition of the many pharmacologists who identified with the society. However, after 35 years, the society was predominantly represented by physiologists, and the pharmacologists had become more closely aligned with the Australian Society of Clinical and Experimental Pharmacologists and Toxicologists (ASCEPT). So, in 2003, the society returned to its name of inception, AuPS.

Over the years, the AuPS has been instrumental in promoting physiology through research, teaching, and knowledge dissemination. A key aim of AuPS has been and continues to be to encourage the representation of all areas of physiological sciences at its meetings. Historical perspectives in our archives do not reflect the challenges that were evident maintaining a unified single society of physiologists in Australia. This is perplexing, given that physiology underpins many other disciplines.

Our archives illuminate the contributions of those who paved the way for us. Without the pioneering efforts of women physiologists such as the late Professor Mollie Hollman, our first female President, the Australian physiology landscape would be vastly different today.

Currently, our council and executive group are led by women, who also constitute at least half of our members, invited speakers and attendees at each annual scientific meeting. This is a reflection of the society's commitment to gender equity, reinforced by our gender equity statement. However, while men and women are equally represented in Australian universities up to the Senior Lecturer (Assistant Professor) level, a significant gender gap exists at senior levels. As a result of this imbalance, Australian government research funding benefits senior male researchers. It is a priority for our society to ensure that all our members have equal opportunities regardless of gender. There has also been little incentive or guidance by the Australian government to ensure that research is designed to identify physiological differences between male and female subjects. It was as recent as July 2024 that the Australian government's national funding body, the National Health and Medical Research Council (NHMRC), released its first recommendations incentivizing researchers to study female populations, or compare male and female populations (https://www.nhmrc.gov.au/research‐policy/gender‐equity/statement‐sex‐and‐gender‐health‐and‐medical‐research). While some research institutes and universities are leading the way with a genuine desire to address this issue, change is slow. In the past 5 years, only 67% of research articles published in the Medical Journal of Australia, the flagship journal of Australian clinicians, included both male and female participants. More concerningly, only 25% of these considered sex a biological variable in their analyses (Ryan et al., 2024).

There is also underrepresentation of indigenous peoples in research studies. Improving health and social equity for Aboriginal and Torres Strait Islander people is one of Australia's most significant challenges. Aboriginal and Torres Strait Islander people currently have a 10‐year lower life expectancy and 2.3 times the burden of disease compared to non‐Indigenous Australians (Australian Burden of Disease Study, 2022). In a recently published large cardiovascular study, while Indigenous, Aboriginal, and Torres Strait Islander people represented 14% of all participants (the prevalence in the Australian population is about 3%–4%), there was almost no representation of any of these populations in arthritis, diabetes, hypertension, kidney, mental illness, and respiratory disease trials despite the significant representation of these groups in the burden of disease (Umaefulam et al., 2022). In November 2023, the Australian Government announced the establishment of The Indigenous Health Research Fund. The fund will invest $160 million in Indigenous‐led research to tackle health issues facing Aboriginal and Torres Strait Islander people. This includes indigenous‐led research practice and governance, and knowledge translation.

Another challenge is that the medical research sector in Australia has long suffered from inadequate investment, leading to a lack of long‐term job security and a decrease in research workforce. These issues have also contributed to the flight of early‐career, mid‐career, and highly skilled researchers from Australia, and from the sector as a whole, which is also of concern. While the Australian government spent $5.5b in 2020–2021 on medical research, only 0.8% of gross domestic product is business expenditure on research and development (Australian Bureau of Statistics, 2021). Our isolation as a country proved advantageous during the recent worldwide COVID pandemic, but attracting industry investment to our shores remains a challenge. Applications to Australian government funding agencies now require a significant translational component to the research proposal. This has posed difficulties for, and excluded some scientists in Australia who primarily seek funds to understand fundamental mechanisms of medical science.

Ironically, despite significant investment by universities and the pharmaceutical industry in developed countries, many challenges impact the research translation path, including difficulties in progressing through clinical trials to drug approval. It is estimated that the success of clinical drug development at clinical trials may be as low at 10% (Sun et al., 2022). At least 45% of those that fail are due to a lack of clinical efficacy. One can easily argue that a fundamental understanding of the mode of action of the drug is of critical importance. The application of artificial intelligence to high‐performance computing, human cell in vitro experimentation, and mechanistic modeling may offer solutions, but this requires significant data input from physiological parameters to validate the in vitro studies.

Physiologists in Australia, as in other countries will be faced with many challenges in the future including external forces such as environmental, political, and social pressures that will shape the way we undertake research. While some may question the relevance of physiology as a discipline, when the Australian Nobel Prize winner Professor Barry Marshall ingested Helicobacter to prove his hypothesis that the bacteria caused ulceration of the stomach, he was practicing the principles of the “Koch's postulates.” Mr Heinrich Hermann Robert Koch who described the postulates would assert that the question of disease causation cannot be resolved without a physiologist!

## FUNDING INFORMATION

We acknowledge the support of a grant from Woodside Energy Ltd, National Health and Medical Research Council of Australia Ideas Grant 2004282. LH is the Wesfarmers, UWA‐VCCRI Chair in Cardiovascular Research at The University of Western Australia.

## CONFLICT OF INTEREST STATEMENT

The authors have no conflicts of interest to declare. Robyn Murphy is Immediate Past President Australian Physiological Society, Séverine Lamon is current National Secretary and Livia Hool is current President.

## DISCLOSURE STATEMENT

The content of this editorial reflects the authors' individual views and not necessarily the official positions of AuPS.

## ETHICS STATEMENT

None.
